# The immunopathology of systemic sclerosis

**DOI:** 10.1007/s00281-015-0517-1

**Published:** 2015-08-19

**Authors:** Jacob M. van Laar, John Varga

**Affiliations:** Feinberg School of Medicine, Northwestern University, Chicago, IL 60657 USA


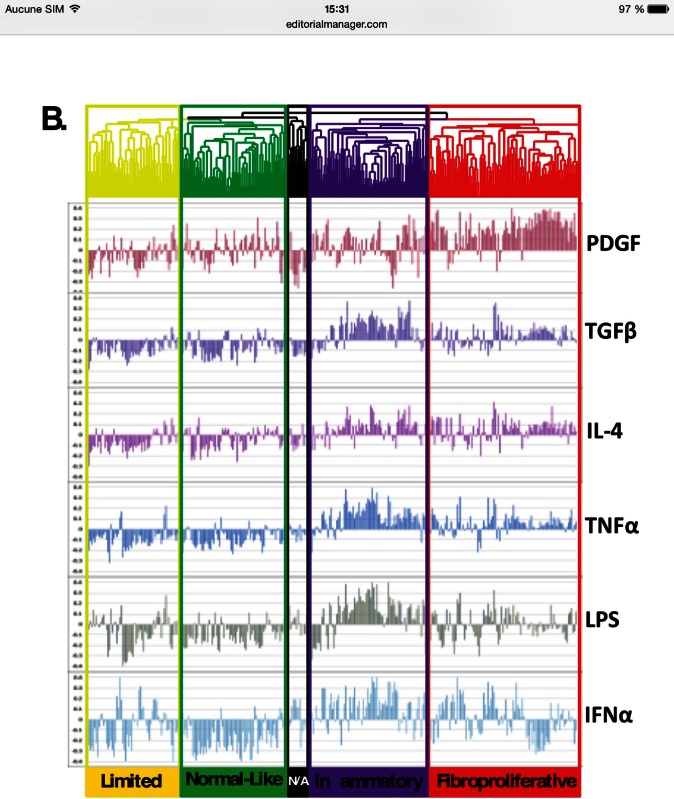


The first sentence in many articles on systemic sclerosis reads something like this: “systemic sclerosis is a rare, autoimmune connective tissue disease of unknown origin affecting the skin and visceral organs, which leads to excess morbidity and premature mortality. Its pathogenesis is characterized by vasculopathy, activation of the immune system and fibrosis.” While these words are true, they ignore the complexity of the biology and the variability in clinical expressions of systemic sclerosis, reflecting a heterogeneous condition. Perhaps systemic sclerosis is better viewed as a syndrome comparable to glomerulonephritis or rheumatoid arthritis rather than a simple disease such as the flu. The present volume on the immunopathology of systemic sclerosis provides ample support for this notion. This is driven, in part, by the advent of unprecedented powerful new laboratory techniques which enable genome-wide association studies, gene-expression studies in blood and skin, multiparameter flow cytometric analyses of cell subsets, proteomics, etc., generating a wealth of data, the analysis of which requires specialist bioinformatic approaches. Yet a sharper focus on the biological processes driving systemic sclerosis is essential if we are to succeed in developing more targeted, hence more effective therapies that result in better outcomes of patients.

Fortunately, the past few years have witnessed major progress in our understanding of the immunopathology of systemic sclerosis, as illustrated by several high-profile publications in the top medical journals [1, 2]. One particularly intriguing theory about the immunological cross-reactivity between malignancy and anti-RNA polymerase III positive systemic sclerosis has come to the fore [1]. According to this theory, malignant cells evoke an immune response as part of a host’s defense to clear the malignant cells. This then may be accompanied by an autoimmune response against specific nuclear antigens present in the malignant cells, which triggers specific autoantibody formation and—somehow—activates other systemic sclerosis pathways, although this part of the story remains to be told. The tight temporary relationship between the malignancy and systemic sclerosis in such cases is suggestive of a common disease mechanism and is in line with other reported epidemiological data indicating a link between malignancy and systemic sclerosis [3, 4]. Autoimmunity may be the price an individual pays to mount an effective anti-tumor response. While this theory has strong appeal, it will be difficult, if not impossible, to prove how this mechanism applies to all cases of SSc, e.g., in SSc patients who do not develop overt malignancy. In the majority of cases, therefore, we remain stuck with the old paradigm that systemic sclerosis just like any other autoimmune disease develops in an (epi)genetically predisposed individual for unknown reasons in the absence of a demonstrable trigger.

Yet these gaps in our understanding of the key decision points involved in disease progression can not obscure the lessons we have learned from meticulously analyzing the aforementioned pathogenetic pathways of systemic sclerosis and (epi)genetic factors. A number of key molecules (eg endothelin-1) involved in vasculopathy have been identified and several targeted therapies are in routine clinical use. As a result pulmonary arterial hypertension (PAH), a previously uniformly fatal complication of systemic sclerosis, has become a treatable chronic condition, although much remains to be learned about the factors driving its progression. Similarly, while it has long been known that innate and acquired immune abnormalities play a role in systemic sclerosis, accumulating data from studies in animal models of inflammation-driven fibrosis and translational research in systemic sclerosis patients have led to a wealth of new therapeutic targets and a flurry of clinical trials. While proof-of-concept trials are a critical step in drug development, ultimately large-scale controlled, randomized trials are necessary to provide the clinical evidence for a drug (or any treatment really) to be shown safe and effective. The history of clinical trials in systemic sclerosis, however, is littered with the carcasses of once promising drugs; think of relaxin, oral collagen, d-penicillamine, imatinib.

We believe that the tide has now turned, and recently completed clinical studies point to better times ahead. An analysis of rituximab-treated systemic sclerosis patients from the European Scleroderma Trial and Research (EUSTAR) registry provided a strong suggestion that B-cell depletion is a valid treatment target [5]. There is a solid scientific rationale for targeting B cells, not only as progenitors of plasma cells which mediate autoantibody production but also as a source of pro-inflammatory cytokines, and actors in antigen presentation. The placebo-controlled phase-2 FASSCINATE trial provided tantalizing data indicating that the prolonged use of tocilizumab may slow down lung and skin disease in patients with early diffuse cutaneous systemic sclerosis [6]. It is hoped that these results can be confirmed in a pivotal phase 3 trial. Interleukin-6 is produced by many cell types implicated in the pathogenesis of systemic sclerosis, including monocytes, endothelial cells, fibroblasts, and B- and T-lymphocytes, is expressed in affected skin in early diffuse cutaneous systemic sclerosis and has profibrotic effects. Serum concentrations have been shown to correlate with the extent of skin thickening and long-term outcome [7]. If confirmed, the beneficial effects of IL-6 neutralization in early diffuse cutaneous systemic sclerosis would underscore a key assumption of its enigmatic pathogenesis, namely that fibrogenesis in certain patients can be disrupted by profound suppression of systemic inflammation. Support for this notion also came from evidence garnered in a recent open-label clinical trial in 15 patients with early diffuse cutaneous systemic sclerosis with a neutralizing antibody against all TGF-ß isoforms. This study showed significant reduction of TGF-ß regulated genes paralleled by an equally rapid and strong improvement of skin thickening [8]. A small placebo-controlled clinical trial with abatacept showed that clinical improvement was associated with modulation of inflammatory pathways in skin [9]. Further large-scale clinical trials with these biologics are needed to confirm their clinical utility and determine the appropriate role of patient stratification.

Perhaps the most compelling evidence for the pathogenic role of systemic inflammation in SSc fibrogenesis so far has come from the international phase-3 ASTIS-trial of 156 patients with early diffuse cutaneous systemic sclerosis who were randomized to treatment with either autologous hematopoietic stem cell transplantation (HSCT) or monthly intravenous pulse cyclophosphamide [10]. With a median follow-up of 5.8 years, the HSCT-treated group showed improved long-term survival, a more rapid and profound improvement of skin thickening, lung function, and quality of life after 2 years, pointing to disease-modifying effects of intensive immunosuppression in poor-prognosis patients.

A main lesson from these clinical trials is that enrichment for patients with early diffuse cutaneous systemic sclerosis is a critical design element in proof-of-concept trials with drugs or biologics targeting immune cells or molecules, and that such trials should be accompanied by a comprehensive analysis of biomarkers and patient and disease characteristics. Notwithstanding the encouraging outcomes of recent clinical trials, much remains to be learned about the nature of the interactions between vasculopathy, inflammation, and fibrosis, and their variable roles in the different patient subsets encompassing the systemic sclerosis spectrum. New technologies are already proving useful in this respect [11].

The present volume hopes to fill the voids with a series of state-of-the-art reviews on different aspects of the immunopathology of systemic sclerosis authored by leading authorities. Taken together, they paint a picture of a great work in progress.
